# Travel Times to Facilities Offering Oral Medication Therapy for COVID-19

**DOI:** 10.1001/jamanetworkopen.2022.49810

**Published:** 2023-01-03

**Authors:** Peter A. Kahn, Xiaohan Ying, Walter S. Mathis

**Affiliations:** 1 Yale School of Medicine, Section of Pulmonary, Critical Care and Sleep Medicine, New Haven, Connecticut; 2 New York Presbyterian Hospital Weill Cornell, New York; 3 Yale School of Medicine, Department of Psychiatry, New Haven, Connecticut

## Abstract

This quality improvement study evaluates access to oral COVID-19 therapeutics within communities in the 48 contiguous states and the District of Columbia.

## Introduction

On December 21, 2021, the US Food and Drug Administration issued an emergency use authorization for nirmatrelvir-ritonavir (Paxlovid; Pfizer) for patients with mild to moderate COVID-19 who are at high risk for progressing to severe disease. Despite the availability of data regarding the location and number of such medications, insufficient data are available regarding the potential access barriers faced by patients. While a recent analysis focused on county-level facility counts, studies have yet to investigate actual accessibility by analyzing travel time to the nearest COVID-19 medication dispenser.^1^ We therefore sought to understand the accessibility of oral COVID-19 therapeutics across the US by examining travel times to locations where these medications are available.

## Methods

The Department of Health and Human Services COVID-19 Public Therapeutic Locator data set was used to identify all active locations at 8 time points in 2022 (January 14, February 16, March 16, April 16, May 16, June 15, July 16, and August 16).^2^ We limited the data set to facilities with oral, outpatient therapies: nirmatrelvir-ritonavir, renally dosed nirmatrelvir-ritonavir, and molnupiravir. As an analysis of aggregate and deidentified public use data, this quality improvement study was not human participant research as defined by the Common Rule and was exempt from review. We followed the SQUIRE reporting guideline.

The population and coordinates of the geometrical center of Census blocks in the lower 48 states and District of Columbia were extracted from the 2020 US Census.^3^ For each block, we determined the 25 geometrically closest COVID-19 therapy access points and computed the shortest 1-way car travel time. Travel times were computed using a local Open Source Routing Machine server built on an OpenStreetMap database of the entire US.^4,5^ A 1-sided *P* = .05 indicated statistical significance.

## Results

**Figure 1.**
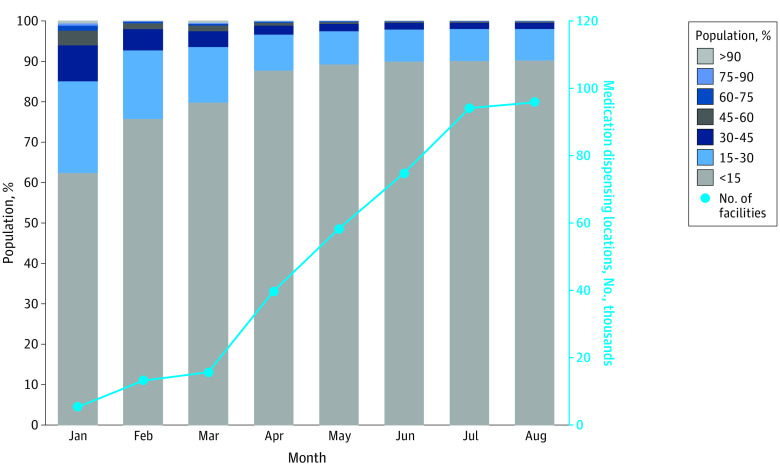
Minimum Travel Time to Oral COVID-19 Medication

**Figure 2.**
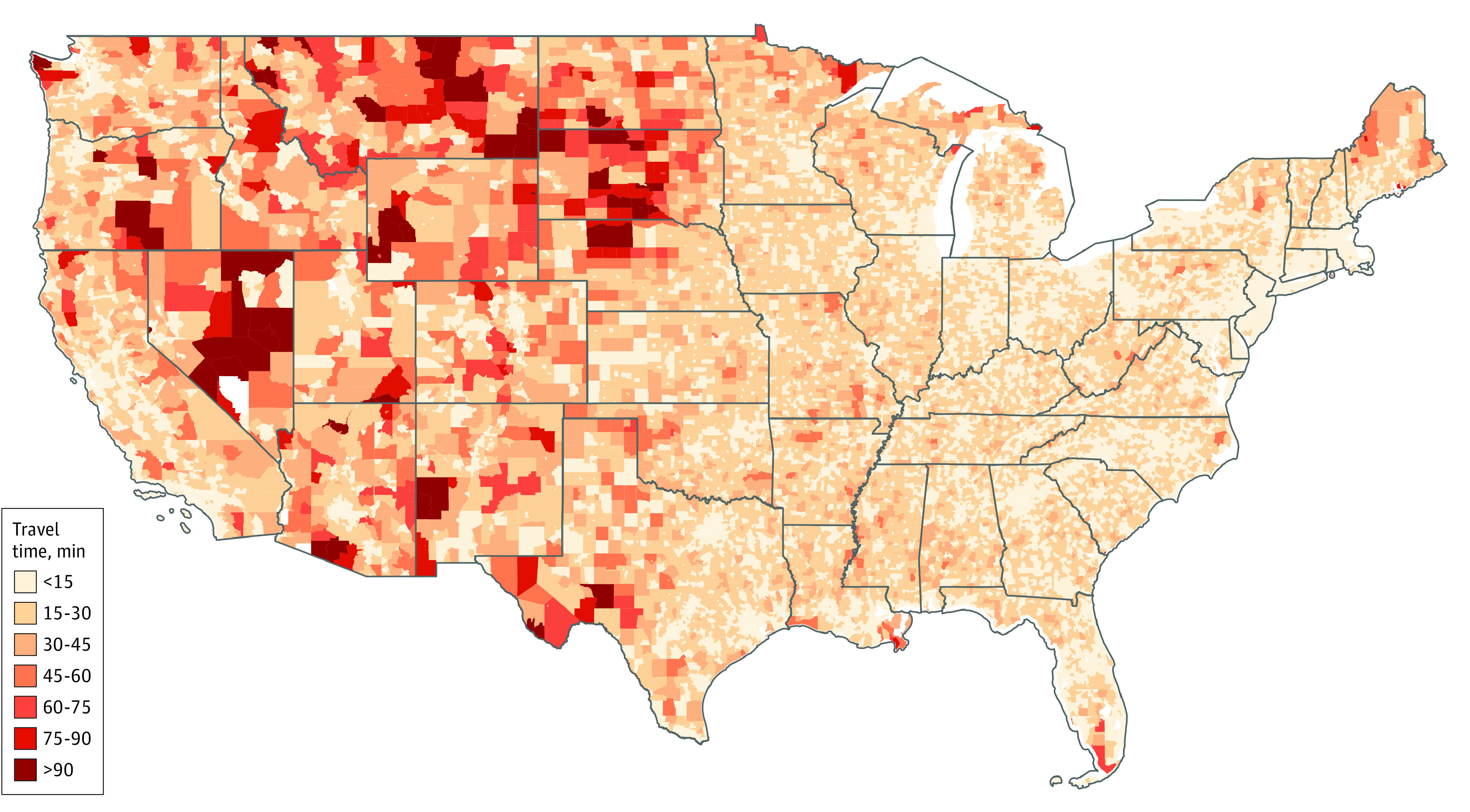
Travel Time by Census Tract to COVID-19 Medication For map representation, population-weighted mean minimum travel times were computed at the Census tract level from constituent blocks.

In the US, the number of locations dispensing oral COVID-19 therapies increased from 5476 in January to 95 797 by August 16, 2022 (Figure 1). In January 2022, only 62% of the population had access to therapy within a 15-minute drive, and 6% were more than 45 minutes away. The number of people living within 15 minutes of the nearest dispenser increased by 11.4% per month (95% CI, 3.6%-19.6% per month; *P* = .02) until April 2022 and has remained mostly unchanged since May when approximately 90% of the population lived within a 15-minute drive from oral COVID-19 therapies (95% CI, −4.1% to 5.0%; *P* = .82). Only 0.3% of the population lived more than 60 minutes away from the nearest dispenser, with South Dakota, North Dakota, Montana, Texas, and Nevada heavily represented (Figure 2).

## Discussion

With over 90% of the US population living within 15 minutes of locations dispensing oral COVID-19 therapies, the national distribution of nirmatrelvir-ritonavir has been successful. However, there are still more than 6 million people living more than 30 minutes and 1.7 million living more than 45 minutes from the nearest dose. This number has not decreased much since May 2022 despite a substantial increase in dispensing locations. Further studies are needed to evaluate these access disparities.

This study has limitations. First, we used only travel time as a benchmark for access to oral therapies. While we believe travel time provides a more accurate perspective than distance, many other factors, such as availability of private vehicles, availability of public transit, functional status, and prescribing patterns, can further restrict access. Second, we potentially overestimated access because it is possible individual pharmacies may not always have sufficient doses on hand. Third, traffic was not included in the analysis, potentially underestimating travel times.

Policy makers and health care systems need to continue to prioritize high-density areas when distributing antiviral supplies, and alternative arrangements should be made for those living in areas without ready access. Strategies may include offering delivery services, including mail, or establishing alternative local access points to ensure oral therapeutics are available to all who may need them.

## Notes

Supplement 1.
Data Sharing Statement
