# Supplementary material for: Travel Times to Facilities Offering Oral Medication Therapy for COVID-19
Source: JAMA Netw Open. 2023 Jan 3;6(1):e2249810. doi: 10.1001/jamanetworkopen.2022.49810 (PMC9857170; doi:10.1001/jamanetworkopen.2022.49810)
Supplement: Supplement 1. — Data Sharing Statement [file jamanetwopen-e2249810-s001.pdf]

Data Sharing Statement

Kahn. Travel Times to Facilities Offering Oral Medication Therapy for COVID-19. *JAMA Netw Open*. Published January 03, 2023. doi:10.1001/jamanetworkopen.2022.49810

Data
Data available: No

Additional Information
Explanation for why data not available: Data used are freely available from public sources - https://healthdata.gov/Health/COVID-19-Public-Therapeutic-Locator/rxn6-qnx8
